# Power profiling and the power-duration relationship in cycling: a narrative review

**DOI:** 10.1007/s00421-021-04833-y

**Published:** 2021-10-27

**Authors:** Peter Leo, James Spragg, Tim Podlogar, Justin S. Lawley, Iñigo Mujika

**Affiliations:** 1 Division of Performance Physiology & Prevention, Department of Sport Science, University Innsbruck, Innsbruck, Austria; 2 Health Physical Activity Lifestyle Sport Research Centre (HPALS), University of Cape Town, Cape Town, South Africa; 3 Faculty of Health Sciences, University of Primorska, Izola, Slovenia; 4 Department of Automatics, Biocybernetics and Robotics, Jožef Stefan Institute, Ljubljana, Slovenia; 5 Department of Physiology, Faculty of Medicine and Nursing, University of the Basque Country, Leioa, Basque Country Spain; 6 Exercise Science Laboratory, School of Kinesiology, Faculty of Medicine, Universidad Finis Terrae, Santiago, Chile

**Keywords:** Racing, Training, Analysis, Performance, Prediction, Power output

## Abstract

Emerging trends in technological innovations, data analysis and practical applications have facilitated the measurement of cycling power output in the field, leading to improvements in training prescription, performance testing and race analysis. This review aimed to critically reflect on power profiling strategies in association with the power-duration relationship in cycling, to provide an updated view for applied researchers and practitioners. The authors elaborate on measuring power output followed by an outline of the methodological approaches to power profiling. Moreover, the deriving a power-duration relationship section presents existing concepts of power-duration models alongside exercise intensity domains. Combining laboratory and field testing discusses how traditional laboratory and field testing can be combined to inform and individualize the power profiling approach. Deriving the parameters of power-duration modelling suggests how these measures can be obtained from laboratory and field testing, including criteria for ensuring a high ecological validity (e.g. rider specialization, race demands). It is recommended that field testing should always be conducted in accordance with pre-established guidelines from the existing literature (e.g. set number of prediction trials, inter-trial recovery, road gradient and data analysis). It is also recommended to avoid single effort prediction trials, such as functional threshold power. Power-duration parameter estimates can be derived from the 2 parameter linear or non-linear critical power model: *P*(*t*) = *W*′/*t* + CP (*W*′—work capacity above CP; *t*—time). Structured field testing should be included to obtain an accurate fingerprint of a cyclist’s power profile.

## Introduction

Since the invention of the first mobile power meter for cycling in the late 1980s training and racing with this tool has become standard practice in multiple cycling disciplines including road, track, mountain bike, cyclo-cross, bicycle motocross (BMX) and triathlon. Mechanical power output measured by strain gauges, most commonly mounted in the bike’s crank spindle, crank arm or pedal spindle and connected to a head unit mounted in the handlebar allows power output data to be accurately recorded in field conditions in real time (Maier et al. 2017). This enables an in-depth analysis of a cyclist’s mechanical power output during training and/or competition, and the assessment of an athlete’s endurance capacity outside of a laboratory setting (Passfield et al. 2017).

These aforementioned technological innovations have allowed applied scientific research to be undertaken in cycling, including real-time measurements of internal (e.g. heart rate) and external (e.g. power output) workloads (van Erp and de Koning 2019; Mujika 2017; Muriel et al. 2021; Padilla et al. 2000; Padilla et al. 2008). This in turn allows the demands of racing to be described (Ebert et al. 2005, 2006; van Erp et al. 2021b; Menaspà et al. 2015; Menaspà et al. 2013; Vogt et al. 2007b), training/racing performance analysis to be conducted (Leo et al. 2021c; Lucia et al. 2001; Mujika and Padilla 2001; Pinot and Grappe 2011) and training prescription to be quantified (Leo et al. 2020; Sanders et al. 2020; Sanders and Heijboer 2019a).

Power profiling in cycling is most commonly defined as the assessment of field derived power outputs, i.e. values obtained during training and racing (Coggan 2003; Leo et al. 2020). Power profiling can be used for the tracking of longitudinal changes in performance and race analysis (Leo et al. 2021b). There is a growing interest in the theoretical and practical implications of power profiling. However, to date, there is no consensus on what constitutes the best practice for power profiling, especially given that there are numerous methodological issues and approaches. Therefore, the aim of this narrative review is to present and discuss existing practices and methods, their implementation, interpretation, and practical applications, provide recommendations to unify power profiling approaches for both practice and research, and suggest future directions for research.

## Measuring power output

Before analysing power output data, it is important to understand how power output is measured during cycling and any associated methodological issues. In cycling, when a force is created by the muscles and applied perpendicular to the bicycle crank arm, one crank arm revolution creates two angular impulses (one per leg); this results in forward drive. Optimal force production, and as a result optimal forward drive, is a complex interplay of innervation, muscle recruitment patterns, the contractile function of muscle as well as an elastic tendon–muscle interaction and metabolic processes occurring in these tissues. The properties of force generation are often described using physics expressions such as mean torque or mean power output; the former describing the force and the latter the amount of work produced in a given time (Winter et al. 2016). Power output is often expressed as a steady-state value (e.g. 100 W), but this value is a product of many impulses over a given period of time or a given proportion of the pedal stroke. Some have argued that ‘mean power output’ is therefore a more accurate descriptor (Winter et al. 2016). Notwithstanding the validity of this argument, for the purposes of this review the authors will employ the customarily used term ‘power output’ throughout. However, it should be noted that power output does not include the energy used to accelerate the cyclist’s limbs nor forces applied in non-propulsive directions.

Mechanical (or external) power output can either be measured by strain gauges or calculated mathematically (Maier et al. 2017; Martin et al. 1998). Depending on the position of the strain gauge (e.g., pedal spindle, crank, bottom bracket), the recorded power output is expected to deviate slightly as some energy is lost via drivetrain inefficiencies (Coyle et al. 1991; Maier et al. 2017; Martin et al. 1998). This highlights that power output values derived from different strain gauge positions may not be comparable. Likewise, different power meter brands and models have different levels of trueness and precision. Maier et al (2017) found that while on average commercially available power meters record at a trueness of − 0.9 ± 3.2% some units will deviate by more than 5%. The authors also reported that some power meter brands have significantly greater precision than others.

On average Maier et al. (2017) found that the smallest worthwhile change for the accuracy of commercially available power meters was 1.1–2.8%. This implies that any performance improvements of less than 1.1% cannot be accurately quantified by commercially available power meter devices. However, this value may differ from brand to brand and model to model. Validation studies have been conducted for most commercially available power meters, but there is no agreed-upon gold standard to which power meters should be compared. Therefore, researchers and practitioners should take note of the comparative measure when assessing the validity of any power output measuring device. We draw the reader’s attention to the aforementioned study by Maier and colleagues (2017) for a broader discussion of the methodological issues surrounding power meter validation. To ensure high data quality the authors strongly recommend accurate calibration according to the manufacturer’s recommendations prior to the collection of any power meter data. Additionally, dynamic (Gardner et al. 2004), static (Wooles et al. 2005), and day-to-day calibration, known as ‘zero-offsetting’ are all recommended before data derived from power meters are used for power profiling purposes.

## Methodological approaches to power profiling

Numerous methodologies have been applied in the field of power profiling. The most basic of these is simply the reporting of average power output values for a given race or event (Ebert et al. 2005; Vogt et al. 2007a, b). While this is the starting point in understanding the demands of a given event, it fails to fully utilise the full potential of power profiling. Another disadvantage is that unless data are derived from cyclists with differing performance levels within an event, this approach does not provide any information on the demands of peak performance, instead it merely describes the demands of participation.

A more advanced approach is to describe the power output by time at a given intensity. This approach is normally described as ‘binning’. Binning is where each power output value is categorized into a bin; each bin represents a range of intensities (for example 100–200 W). The resulting categorization of each output value can then be expressed as either total cumulative time in each bin or as a percentage of total time. (Abbiss et al. 2010; Ebert et al. 2006; Leo et al. 2021b; Metcalfe et al. 2017). Typically, but not always, the bins are defined by normalizing the power output to body mass (for example 4–5 W kg^−1^). However, the suitability of this approach can be questioned; for example, in some events aerodynamic drag is a far more important factor than body mass (Pringle et al. 2011). Besides scaling power output relative to the frontal area (Padilla et al. 1999), to the best of the authors’ knowledge no studies have been published where the bins represent ranges of power output values normalised to aerodynamic drag (W CdA^−1^).

Binning has advantages in that it can describe the range of intensities that are required to compete or perform in a given event. Typically, cycling events are not completed at a fixed power output; instead, power output is stochastic in nature, even in individual time trials (Gordon 2005). Whilst binning allows the total time at different intensities to be described, there are weaknesses with this approach. Firstly, the choice of the range of intensities for a given bin will influence the results. Often arbitrary bins are chosen, based on a given power output normalized to body mass, for example 5.9–7.9 W kg^−1^. If the range of intensities is too wide the granularity of the power output data cannot be captured. Another problem is that binning gives no insight into the length of individual efforts. The cumulative time in each power output bin may represent one long effort or multiple short efforts. Finally, if arbitrary bins are used then it may be that the range of intensities covered by a single bin includes power outputs that are both sustainable and unsustainable from a physiological point of view. A solution to this problem is to use physiological thresholds to define the bins (Abbiss et al. 2010; Passfield et al. 2013). For example, the submaximal physiological thresholds that define the exercise intensity domains could be determined during laboratory testing and used to define the bins. While this approach does give a greater insight into the physiology of a given event for individual athletes, problems occur when data from multiple athletes are amalgamated, as the bins, while representing consistent physiological responses, do not necessarily represent the same absolute or relative power output for all athletes.

**Fig. 1:**
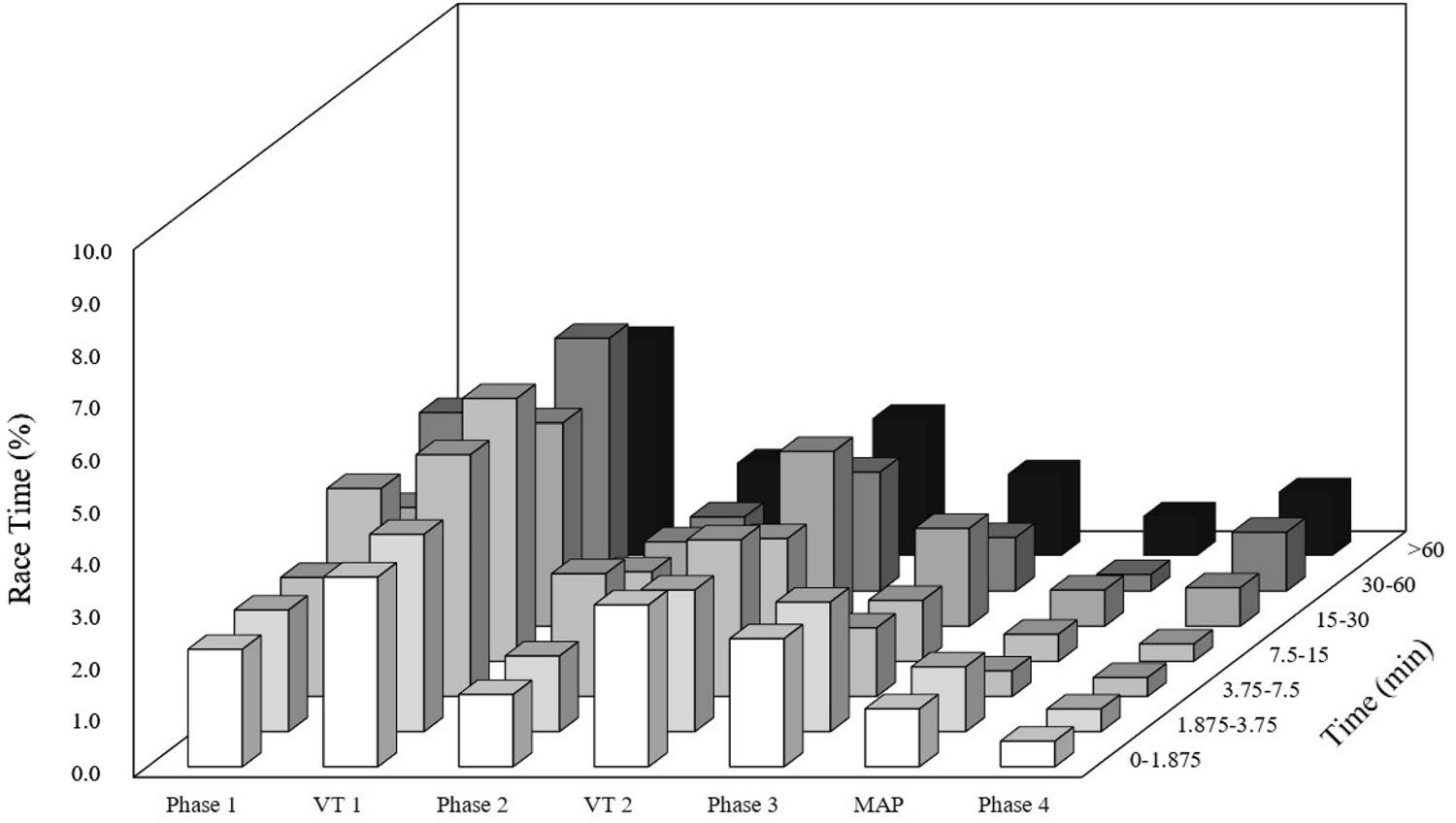
EVA—exposure variation analysis in the final hour of a race in six U23 cyclists (*N* = 6)

As previously mentioned, one of the main problems with binning is that duration of individual efforts are not represented within the data. However, there is a small body of work that uses exposure variation analysis (EVA) to try and overcome this limitation (Abbiss et al. 2010; Passfield et al. 2013). This approach uses a two-bin system; one set of bins is used in the traditional manner to describe the intensity. Bins can be associated with either arbitrary values or physiological thresholds. The second set of bins is used to describe the duration of individual efforts. Here arbitrary durations are used, for example 0–5 s, 5–10 s or > 1 min. The intensity bins are plotted on the x-axis, the duration of individual efforts is plotted on the z-axis and the percentage of total race time is plotted on the *y*-axis (see sample data in Fig. 1).

Whilst the exact power output of individual efforts is still not displayed, EVA is a very powerful tool to show the pacing strategy and stochastic nature of power output in a given event. This approach may be especially powerful to describe events where lots of short submaximal sprints are interspersed by periods of recovery, for example cyclo-cross or Olympic cross country mountain biking. EVA is an effective way to describe the duration of efforts and recovery bouts. This information can be valuable for coaches and practitioners when prescribing interval training sessions to replicate the demands of an event.

A major limitation of the approaches discussed thus far is that they fail to describe power outputs for individual efforts. To do this the mean maximal power output (MMP) approach can be used (van Erp and Sanders 2020; Puchowicz et al. 2020; Quod et al. 2010; Vogt et al. 2007b). MMP values represent the highest average power that was recorded for a given (arbitrary) duration, during an event. For example, the highest average power output recorded over a 5 min duration in a race would be the 5 min MMP. Such MMP data are very valuable because they make it possible to identify the power output and duration that a cyclist is required to produce to be competitive in an event. For example, MMP data analysis shows that a top male general classification contender in a grand tour is required to produce 5.8 W kg^−1^ for 20 min on key mountain climbs (van Erp et al. 2020a, b). For coaches and practitioners this is very valuable information.

There are however some fundamental issues with MMP data. Firstly, it is not known if the recorded MMP values were derived from a maximal effort. This contrasts with values derived from formal testing where the maximality of an effort can be verified. For example, in a traditional laboratory incremental graded exercise test (GXT) a given perception of effort and respiratory exchange ratio need to be obtained for the test to be considered maximal in nature and therefore a valid maximum oxygen uptake ($\dot{V}$O_2max_) value to be obtained (Jones et al. 2016). It is hypothesised that almost none of the MMP values derived from races are maximal in nature. If a rider were to produce a maximal effort at any point other than at the finish of a race, it may compromise their ability to subsequently follow their competitors in bunch events or compromise their pacing strategy in individual events (Leo et al. 2021b, c). Secondly, MMP data from a specific (arbitrary) duration could be the result of the bracketing of a subsection of a longer effort, or a shorter duration effort and a subsequent recovery (Leo et al. 2021a, b). For example, it is very unlikely that a 5-min MMP value derived from a race represents a maximal effort of exactly 5 min in duration. As a result, there is a high probability of an inherent underestimation of maximal power output when using MMP values alone. MMP data are only indicative of what a cyclist did, not what the cyclist is capable of.

Another issue with MMP data in research is that there is no agreed-upon set of (arbitrary) durations that are being applied. This means that when trying to compare data from various studies coaches and practitioners cannot perform like for like comparisons. This situation has improved somewhat as research groups have started to incorporate a wider range of MMP durations from ~ 5 to ~ 1800s. This allows a power-duration curve to be developed using the MMP values, allowing for some comparisons between studies. A final issue with MMP analysis is that it may not actually define ‘race winning efforts’. Recent work by Leo and colleagues (2021b) and van Erp and colleagues (2021a; b) showed that the power output that cyclists produce falls throughout an event; and that MMP values are not predictive of race performance. Instead, it is the power output that riders produce at key moments in the race that is predictive of performance. For example, in the case of a sprinter in road cycling it is the power that they can produce in the final moments of the race that is important, but this is not necessarily the same as their 10 s MMP. This means that MMP analysis may be missing the very efforts that it is trying to identify. To better identify these race-winning efforts an approach has been taken in research whereby the event is broken down into segments and MMP values in each segment have been reported (van Erp et al. 2021a; Leo et al. 2021b; Sanders and van Erp 2021). To date, these segments have been defined via accumulated work, either absolute values or normalized to body mass, for example MMP values after 2.500 kJ of work. However, this approach, which has thus far only been applied in road cycling has introduced some further limitations. Road cycling is a team sport in which riders perform individual tasks such as sheltering a team leader or collecting nutrition from a following car. It is not the goal of every rider to try and win the race. Therefore, the reported decrease in MMP values, as accumulated work increases, may partially be a product of the fact that some riders have simply finished their tasks and are therefore no longer producing maximal efforts.

To alleviate the problem of arbitrary MMP durations not matching actual effort durations, some studies have selected specific sections of the event and identified power output exclusively in that section (Jobson et al. 2008; Leo et al. 2021c; Padilla et al. 2008). For example, Leo and colleagues (2021a; b) looked at MMP values exclusively on classified climbs. This approach, while potentially beneficial in certain circumstances, does require researchers to identify the key moments in races for analysis. While this may be possible for some events, such as a road race stage that starts out flat and concludes with a mountain top finish, it is not always possible to accurately identify the key moment in a race. A possible solution to this is to seek the input of athletes when identifying the key periods in the race. Whilst an attractive proposition, to the best of the authors’ knowledge this approach has not been used in published research.

As mentioned before, the major issue with MMP analysis is the uncertainty surrounding whether an effort was maximal in nature, and whether the MMP duration is equal to the effort duration. To counter this problem, the authors recommend using power output values derived from formal testing to provide a comparative measure to MMP values. This approach has particular benefits for coaches and practitioners as comparisons between MMP data and formal testing data can be used to monitor changes in the power profile; and if a rider records a MMP value which exceeds the prediction from formal testing a new formal performance test can be scheduled. This is particularly useful when analysing performance in timed events where the in-competition power output and event duration can be compared to the theoretical power-duration relationship. This example highlights the importance of developing a power-duration relationship rather than simply using standard duration performance tests, as the likelihood of the test and competition durations being identical is low. For methodological issues surrounding the development of theoretical power duration relationships please see the section ‘Deriving a power-duration relationship’ below.

Unfortunately, this approach (i.e. comparing MMP against a pre-established theoretical power-duration relationship derived from prior formal testing) was only undertaken by a few research groups (Leo et al. 2020; Leo et al. 2021b; Nimmerichter et al. 2020; Quod et al. 2010). However, all research has shown good to very good agreement between power output values from formal testing and MMP values. Of particular interest is work by Leo and colleagues (2020, 2021a) that shows the formal testing values are only predictive of race performance for a 6-month period before formal re-testing is required.

### Methodological issues

Thus far we have discussed methodological approaches in power profiling, however, there are also methodological issues that are pertinent to all approaches. Recorded power output values can be highly influenced by the topography of the event (Padilla et al. 2000, 2008; Sanders and Heijboer 2019a), differences between single day and multi-day stage racing (van Erp and de Koning 2019; van Erp and Sanders 2020; Lucía et al. 2003) and race category (Sanders and van Erp 2021). In professional road cycling race category was found to influence power output: higher power outputs over shorter durations (< 2 min) were reported in lower-ranked races, and higher power outputs over longer durations (> 10 min) were observed in races with higher difficulty. Another important consideration when performing power profiling are environmental factors. Altitude, temperature, and humidity can all influence the power output athletes can produce. Therefore, from a research perspective the authors recommend that the environment and race conditions should be reported whenever possible.

Recent research has also shown that power profiling analysis conducted exclusively on either training or racing data produces different results in the same participants (Leo et al. 2020). This is an important factor and further highlights the need to provide adequate information on the context in which any power profiling data were collected.

Finally, in competition settings, alongside the aforementioned issues surrounding team roles there is an influence of other team-mates and competitors on power output due to drafting, which lowers the power output requirement for a given speed (Ouvrard et al. 2018, van Druenen and Blocken 2021). Research has also suggested that competition may influence the pacing strategy adopted by cyclists (Bossi et al. 2018).

## Deriving a power-duration relationship

When power output is plotted against time to task failure (TTF) a consistent power-duration relationship emerges (Burnley and Jones 2018). The first researchers to mathematically describe this relationship were Monod and Scherrer (1965) who analysed muscle fatigue during static and dynamic work (knee extension exercise) and created a mathematical model describing the hyperbolic relationship between completed work and TTF. Due to the strong scientific evidence over decades (Burnley and Jones 2018; Jones et al. 2010; Poole et al. 2016) the power-duration relationship can be considered to represent an integrative approach to the limits of tolerable exercise in humans.

From a physiological perspective the power-duration relationship is comprised of four distinct exercise intensity domains; namely, moderate, heavy, severe, and extreme (Burnley and Jones 2007), which are characterised by distinct whole-body physiological responses (Jamnick et al. 2020; Vanhatalo et al. 2016; Whipp 1996). While a complete physiological background on the systemic and mechanistic bases of the power-duration relationship would be beyond the scope of this narrative review, interested readers are referred to the following review articles: Burnley and Jones (2018), Jones et al. (2010), Poole et al. (2016), Poole et al. (2021), Vanhatalo et al. (2016).

**Fig. 2:**
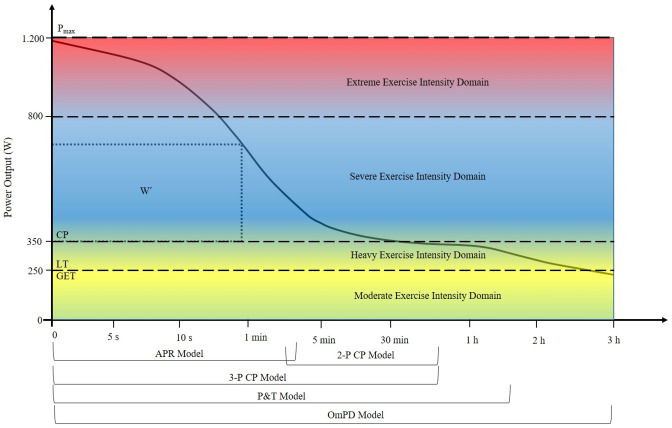
An illustration of the spectrum of physiological responses across the power-duration relationship using arbitrary power output values. *P*_*max*_ 1 s peak power, *W*′ work above critical power, *CP* critical power, *LT* lactate threshold, *GET* gas exchange threshold, *APR* anaerobic power reserve model, *2-P CP* two-parameter critical power model, *3-P CP* three-parameter critical power model, *P&T* Peronnet and Thibault Model, *OmPD* omni power duration model

Various models are available to coaches and practitioners to model the power-duration relationship for use in power profiling (Sreedhara et al. 2019). However, most models only cover a specific section of the power-duration relationship (see Fig. 2).

### Modelling power output in the extreme exercise intensity domain

**Fig. 3:**
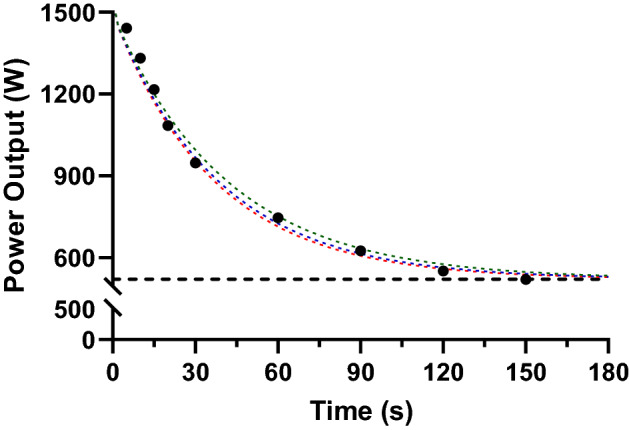
Sample data for the anaerobic power reserve model, black dots—record power output over 5, 10, 15, 30, 60, 90, 120 and 150 s durations; horizontal black dashed line:—anaerobic power reserve; green, blue and red dashed lines representing the power duration curve with the rate constant (*k*) of the exponential decline in power output (*k* = 0.024, *k* = 0.026, *k* = 0.027) according to Sanders and Heijboer (2019b)

Previous research (Bundle et al. 2003; Bundle and Weyand 2012; Weyand et al. 2006) has demonstrated that the anaerobic power reserve (APR) is capable of predicting short duration (< 3 min) power outputs within the extreme exercise intensity domain, where $\dot{V}$O_2max_ may not be attained before task failure occurs. The APR approach was initially developed in laboratory settings where the maximum aerobic power (MAP) recorded during a GXT and the maximal power an athlete can produce over one pedal revolution or over one second (*P*_max_) are used as parameter inputs. However, Sanders et al. (2017, 2019b) developed a field testing method where 3 min MMP can be used as a surrogate for MAP. In this approach the time constant (*k*), which can be defined as the rate of the exponential decline in power output (i.e. the reciprocal of the corresponding time constant: *k* = 1/τ), can be varied between values of 0.024–0.027 to best fit the MMP data. This allows for an individualisation of the power-duration relationship modelling, which may provide a better fit (Sanders and Heijboer 2019b) [see sample data in Fig. 3 and Table 1 (Eq. 1)].

**Table 1:** Power-duration models corresponding to the respective exercise intensity domains

Exercise intensity domains	Model	Equation
extreme	Anaerobic power reserve	$P_{\left( t \right)} = P_{{\left( {3 - min} \right)}} + { }\left( {P_{{\left( {max} \right)}} - { }P_{{\left( {3 - {\text{min}}} \right)}} } \right) \times e^{{\left( { - k \times t} \right)}} { }$(1)
extreme and severe	3-parameter critical power model	$t = \frac{{W^{\prime } }}{P - CP} + \frac{{W^{\prime } }}{{CP - P_{\max } }}$ (2)
Severe	2-parameter critical power model	$P_{\left( t \right)} = \frac{{W^{\prime } }}{t} + CP$ (3)
extreme, severe and heavy	Peronnet and Thibault model	$Pmap_{\left( t \right)} = MAP - A \times Ln\left( {\frac{t}{{MAP_{TTF} }}} \right);\quad t > MAP_{TTF}$ (4)
	Omni power duration model	$P_{\left( t \right)} = \frac{{W^{\prime } }}{t} \times \left( {1 - e^{{ - t \times \frac{{P_{\max } - CP}}{{W^{\prime } }}}} } \right) + CP;\quad t \le CP_{TTF}$ $P_{\left( t \right)} = \frac{{W^{\prime } }}{t} \times \left( {1 - e^{{ - t \times \frac{{P_{\max } - CP}}{{W^{\prime } }}}} } \right) + CP - A \times Ln\left( {\frac{t}{{CP_{TTF} }}} \right);\quad t > CP_{TTF}$(5)

Equation 1: *P*_(*t*)_ power output, *P*_*(3-min)*_ 3 min field test, *P*_(max)_ 1 s peak power, *e* base of the natural logarithm (2.718), *k* the rate constant of the exponential decline in power output, *t* time in seconds

Equation 2: *t* time in seconds, *W*ʹ work above critical power, *P* power output, *CP* critical power, *P*_*(max)*_ 1 s peak power

Equation 3: *P*_*(t)*_ power output, *W*ʹ work above critical power, *CP* critical power, *t* time in seconds

Equation 4: *Pmap(t)* power output at maximum aerobic power, *MAP*_*TTF*_ time to task failure at maximum aerobic power, *t* time in seconds, *A* represents a fixed constant for the decline in power output over time, *Ln* natural logarithm to the base of e (2.718)

Equation 5: *P*_*(t)*_ power output, *W*ʹ work above critical power, *CP* critical power, *t* time in seconds, *CP*_*TTF*_ time to task failure at critical power, *A* represents a fixed constant for the decline in power output over time, *Ln* natural logarithm to the base of e (2.718)

**Fig. 4:**
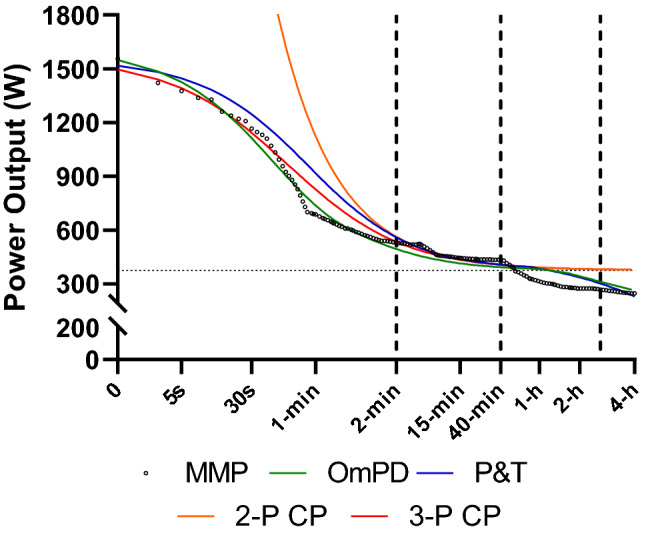
Various power duration modelling approaches applied to the same MMP data. *MMP* Mean Maximum Power, *OmPD* Omni Power Duration model, *P&T* Peronnet and Thibault model, *2-P CP* two-parameter critical power model, *3-P CP* three-parameter critical power model; horizontal dashed line—critical power asymptote; vertical dashed lines represent the approximate transitions between the exercise intensity domains (extreme, severe, heavy and moderate)

Alongside the APR model, power output in the extreme exercise intensity domain can also be predicted using the three-parameter critical power (3-P CP) (Morton 1996), the Peronnet and Thibault model (P&T) (1989) and Puchowicz’s omni power duration model (OmPD) (Puchowicz et al. 2020). It should be noted that in the P&T model, *P*_max_ is provided as a parameter estimate, whereas in the APR model, 3-P CP model and the OmPD model *P*_max_ is required as an input parameter. These different modelling approaches considerably influence power output predictions in the extreme exercise intensity domain (see Fig. 4).

### Modelling power output in the severe exercise intensity domain

Multiple approaches based on the CP concept have been proposed to predict power outputs within the severe exercise intensity domain. Although all CP models are equivalent from a mathematical perspective (i.e. they can be derived mathematically from one another) they produce different statistical parameter estimates for CP and work above CP (W′) (Jones et al. 2010; Muniz-Pumares et al. 2019), and therefore slightly different predictions within the severe exercise intensity domain; particularly at the extremes of the domain. The 3-P CP model (Morton 1996) aimed to overcome these limitations for short duration power outputs toward the upper end of the severe and into the extreme exercise intensity domain by incorporating P_max_ as a model parameter, but it still overestimates power outputs in the moderate exercise intensity domain (see Fig. 4).

### Modelling power output below the critical power

The CP represents the theoretical asymptote of the power-duration curve, suggesting that a given power output is infinitely sustainable. However, this is clearly not the case for real-world performances where exercise at the CP is limited to 20–40 min (Poole et al. 2016). For this reason, previous research (Peronnet and Thibault 1989; Puchowicz et al. 2020) has suggested an exponential decay term below the CP to predict power outputs in the heavy exercise intensity domain (see Fig. 2 and Eqs. 4 and 5). However, these decay terms are not necessarily routed in the underlying physiology of fatigue in the heavy and moderate exercise intensity domains (see Black et al. (2017), Clark et al. (2019) and Amann (2011) for overviews of possible fatigue mechanisms at these intensities). They do however represent the best models to date for estimating exercise tolerance below the CP (see equations in Table 1).

### Choosing a modelling approach

The authors recommend that coaches and practitioners refer to the physiological demands of a given discipline or training modality to guide their choice. They should then select the model that best predicts the power-duration relationship across the range of intensities in which athletes will train and race. For example, the two-parameter CP model (Moritani et al. 1981; Whipp et al. 1982) overestimates both short- and long-duration power outputs outside the severe exercise intensity domain (see Fig. 4), thus potentially limiting its utility. To give some practical examples; power outputs in the team sprint falls exclusively in the extreme exercise intensity domain, whereas power outputs in the individual pursuit falls within both the extreme and severe exercise intensity domains (Gardner et al. 2005). In road cycling a large proportion of the power output falls within the heavy and moderate exercise intensity domains (van Erp and de Koning 2019); however, power outputs in the extreme and severe exercise intensity domains are more important in predicting race performance (Menaspà et al. 2017). Longer duration (ultra) endurance events, for example, ironman distance triathlons (Laursen 2011) or the ‘Race Across America’ (Hulton et al. 2010) fall within the moderate exercise intensity domain, as do extensive training sessions in cycling or triathlon (van Erp et al. 2020b; Laursen 2011). A different modelling approach may be required for each of these examples.

Interestingly some of the aforementioned models are able to predict exercise tolerance in multiple exercise intensity domains. Whilst there is a considerable body of evidence indicating that the physiological responses in each exercise intensity domain is unique (Burnley and Jones 2007), it should be noted that most research is derived from exercise intensities that are not in close proximity to the thresholds that define a given exercise intensity domain. Work by Pethick and colleagues (2020) looking at responses in proximity to the critical torque (CT) during isolated knee extension exercise, a proxy for CP, showed that above the CT participants displayed physiological responses consistent with the severe exercise intensity domain. Likewise, slightly below the CT physiological responses associated with the heavy exercise intensity domain were recorded. Another pertinent example is that research has shown that although the $\dot{V}$O_2_ slow component is a defining characteristic of the heavy exercise intensity domain, a variant of the slow component, albeit smaller in magnitude, also occurs in the moderate exercise intensity domain (Davies and Thompson 1986). Whilst a proportion of the change in $\dot{V}$O_2_ uptake may be due to a shift in substrate utilisation, this change wouldn’t account for the entire increase in $\dot{V}$O_2_, suggesting altered or additional muscle recruitment (Burnley and Jones 2018). Together, these findings suggest that rather than each exercise intensity domain inducing distinct physiological responses, there is instead a spectrum of responses across the power-duration relationship (see Fig. 2). Indeed, this would explain why the power-duration curve is smooth in nature and doesn’t contain ‘turn-points’ as would be expected if the thresholds between exercise intensity domains were indeed ‘hard’ in nature. It may also explain why some of the aforementioned models are able to predict exercise tolerance across intensities in multiple exercise intensity domains (Fig. 2 and Table 1).

## Combining laboratory and field testing

Both laboratory and field testing have been used in isolation and in conjunction with each other to investigate physiological and performance capacity in cycling (Gardner et al. 2007; Jobson et al. 2009; Jones and Vanhatalo 2017; Lucia et al. 2001; Paton and Hopkins 2001).

In cycling, the most commonly reported measures from laboratory testing include peak power output from sprinting or graded incremental exercise tests, $\dot{V}$O_2max_, %$\dot{V}$O_2max_, MAP, fractional utilization of MAP, first and second lactate or ventilatory thresholds, maximum lactate steady state and cycling efficiency (Laurent et al. 2007; Lucia et al. 2000; Mujika and Padilla 2001). Although good agreement exists between some of these laboratory measures and cycling performance, none of the aforementioned physiological variables can be used to create a power-duration relationship as recommended by the authors for the purposes of power profiling.

As demonstrated before, a critical component of the power-duration relationship is the border between the heavy and severe exercise intensity domains; power outputs at which a steady state can and cannot be achieved (Poole et al. 2016; Poole et al. 1988). The physiological boundary between these domains has been most associated with endurance performance (Burnley and Jones 2007; Poole et al. 1988). For a long time, the maximum lactate steady state (MLSS) was considered as the gold standard for this boundary (Billat et al. 2003; Keir et al. 2015; Kilding and Jones 2005). However, recent work (Galán-Rioja et al. 2020; Jamnick et al. 2020; Jones et al. 2019; Nixon et al. 2021) has suggested that CP better estimates the maximal metabolic steady state, the highest power output where a steady state in the oxygen uptake ($\dot{V}$O_2_) response can still be observed, despite increasing blood lactate values (Bräuer and Smekal 2020). There is still some debate as to which method (if any) is superior for differentiating between metabolic steady state and non-steady state exercise, and whether both MLSS and CP can actually be used interchangeably (Jones et al. 2019; Keir et al. 2015; Nixon et al. 2021; Poole et al. 1988).

In applied settings, it has been suggested that an alternative approach, namely the functional threshold power (FTP), can be used as a surrogate for the maximal metabolic steady state: (Mackey and Horner 2021). FTP was first described as the cycling power output that can be sustained for one hour in a “quasi physiological steady-state” (Bassett et al. 1999; Coggan 2003; Mackey and Horner 2021). FTP is therefore a surrogate of the 60 min MMP. It has been proposed that FTP can also be predicted either by taking 95% of the power output in a 20-min maximal field test (Borszcz et al. 2018; Morgan et al. 2019; Valenzuela et al. 2018) or by taking 90% of the power output in a 8-min maximal field test (Sanders et al. 2020); the former being commonly used (Valenzuela et al. 2018). In contrast to CP and MLSS, where multiple determination trials are required, FTP can be predicted from a single trial and is, therefore, less time consuming. This time efficient approach may explain why the concept has been widely adopted in cycling (Mackey and Horner 2021). However, whilst CP and MLSS can be considered as estimates of the maximal metabolic steady state (Keir et al. 2015; Poole et al. 1988), this cannot be confirmed for FTP (Morgan et al. 2019). Whilst both MLSS and FTP are single-parameter estimates, the CP concept can be used to predict TTF for a range of power values within the severe exercise intensity domain and provides an estimate of the border between metabolic steady state and non-steady state exercise. The same cannot be said for either MLSS or FTP, which can only predict a single point on the power-duration relationship, or a border between exercise intensity domains, but not TTF for a range of power output values.

Physiologically speaking, CP has been shown to represent the highest power output at which there is no progressive derangement in the muscle metabolite milieu (Burnley and Jones 2018); however, instead of a ‘hard’ border, the CP represents a phase transition between the heavy and severe exercise intensity domains (Pethick et al. 2020). Mitchell and colleagues (2018) also reported a strong relationship between CP and muscle capillary density, underpinning the aerobic component of CP. Similarly, Vanhatalo et al. (2016) demonstrated that CP was strongly associated with the percentage of highly oxidative type I muscle fibres. Above CP, in the severe exercise intensity domain a non-metabolic steady state occurs, characterized by a reduction in intramuscular creatine phosphate stores, continuously increasing concentrations of inorganic phosphate, hydrogen ions and blood lactate, which are all associated with a reduced contractile function of the working muscle (Allen et al. 2008; Burnley and Jones 2007, 2018; Jones et al. 2010; Poole et al. 2016, 1988).

Although a strong relationship exists between FTP and CP estimates (Denham et al. 2020; Karsten et al. 2020; Morgan et al. 2019, Mackey and Horner 2021), and FTP and MLSS (Borszcz et al. 2019), the cited studies have demonstrated that the limits of agreement between parameters are too large for them to be used interchangeably. This questions the relevance of FTP (Borszcz et al. 2018; Karsten et al. 2020; Morgan et al. 2019; Valenzuela et al. 2018). Furthermore, Borszcz and colleagues (2018) demonstrated that the 95% of 20 min power output overestimates 60 min power output, and recommended that 20 min power output alone should be used for training prescription and performance monitoring, rather than trying to make estimates of 60 min power output (i.e. FTP). After all, both 20 and 60 min power output are arbitrary in nature. However, whilst FTP might represent an arbitrary value, rather than a physiological threshold, it may still have practical utility in terms of informing the training process (Valenzuela et al. 2018). However, to the best of the authors’ knowledge no studies exist that compare performance outcomes when prescribing training based on different concepts, i.e. FTP, CP and MLSS.

That said, for the reasons outlined above the authors consider CP the most useful concept in terms of deriving a power-duration relationship, and therefore recommend the use of the CP concept in the field of power profiling.

## Deriving the parameters of power-duration modelling

There is currently no consensus on how best to derive the parameters that are needed to model the power-duration relationship; namely *P*_max_, CP and *W*′. Likewise, there is considerable debate on which mathematical model should be used to derive CP and *W*′ (Maturana et al. 2018; Muniz-Pumares et al. 2019; Nimmerichter et al. 2020).

Traditionally, performing three to five prediction trials between 2 and 15 min of duration (Karsten et al. 2015; Maturana et al. 2018; Muniz-Pumares et al. 2019) allows CP and *W*′ to be derived through weighted least square or geometric mean linear and nonlinear regression analysis (Vinetti et al. 2017; Vinetti et al. 2020). Prediction trials shorter than 2 min do not ensure the attainment of $\dot{V}$O_2max_ (i.e. they fall outside the severe intensity domain) (Hill and Smith 1994; Maturana et al. 2018; Muniz-Pumares et al. 2019; Nimmerichter et al. 2020), while prediction trials longer than 15 min are not recommended due to the influence of glycogen depletion and psychological factors (i.e. motivation) (Karsten et al. 2015; Maturana et al. 2018). To avoid any skewness during the mathematical modelling and reduce errors in the calculation of CP and *W*′ the shortest prediction trial should last between 2 and 5 min and the longest prediction trial between 12 and 15 min (Karsten et al. 2015; Maturana et al. 2018; Muniz-Pumares et al. 2019). Inter-trial recovery between prediction trials should be set to a minimum of 30 min during a single visit or 24 h during multiple days (Karsten et al. 2017). The benefit of multiple days if that any fatigue induced by the initial prediction trial does not affect the subsequent one, but possible error due to day-to-day variation in power output is introduced.

**Fig. 5:**
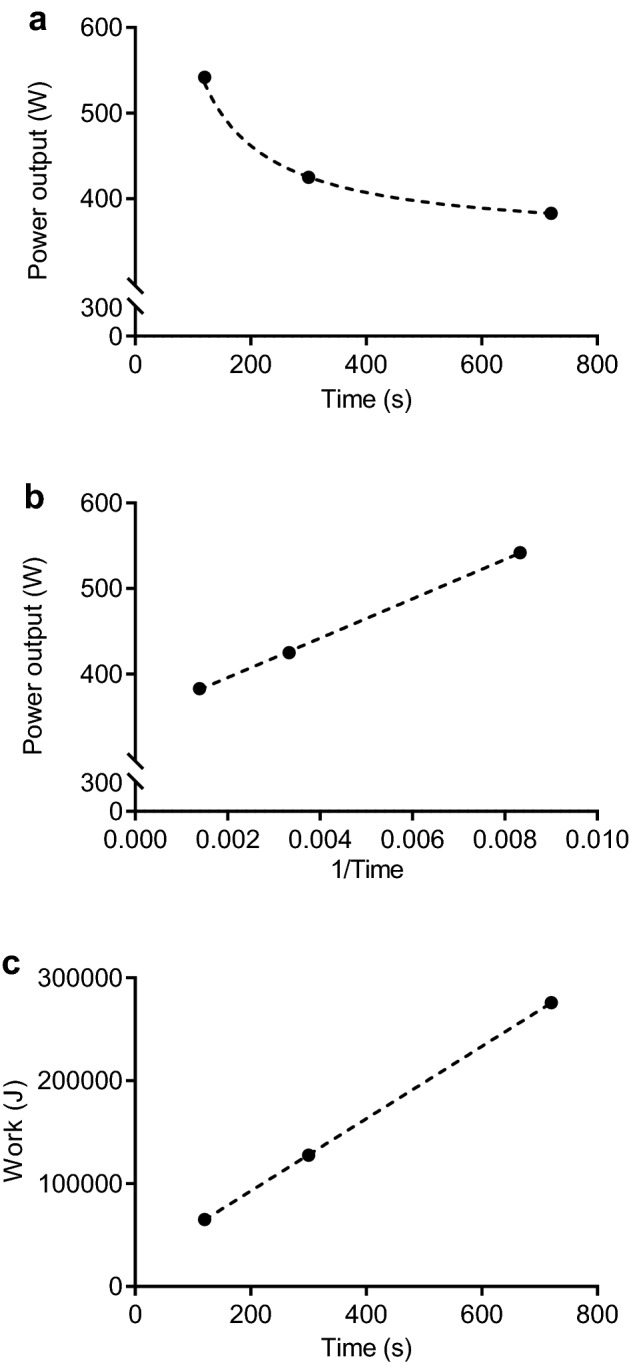
Graphical illustration of the power-duration relationship for the hyperbolic (**a**), inverse of time (**b**) and linear work time (**c**). Model adopted from Clarke and Skiba (2013)

Once the performance trials have been completed the respective power output and trial duration values can be used to derive CP and *W*′. Computing CP and *W*′ estimates from a nonlinear two- or three-parameter models requires access to statistical software to perform a weighted least square or geometric mean regression analysis (Vinetti et al. 2017, 2020). To simplify this process for coaches and practitioners there are two options available to linearize the hyperbolic power-duration relationship (see Fig. 5). Practitioners can either use a) the linear work time CP model (see Eq. 3 and Fig. 5c or b) the linear power inverse of time CP model (see Eq. 3 and Fig. 5b), where CP and *W*′ can be derived as the slope and intercept of the linear relationship (Clarke and Skiba 2013; Sreedhara et al. 2019). All mathematical models from Fig. 5 provide a high accuracy for the model fit, but there is a possibility that the power-duration parameter estimates (CP and *W*′) diverge somewhat depending on which fitting method is used (Muniz-Pumares et al. 2019). As a result, Hill (1993) suggested that the best fit mathematical model could be more objectively selected, where the model producing the lowest standard error of the estimate (SEE) should be the preferred way to derive CP (Hill 1993; Muniz-Pumares et al. 2019).

CP and *W*′ parameter estimates can also be derived using only two prediction trials (Parker Simpson and Kordi 2017). While this can be seen as a time-efficient testing protocol, the limitation of this approach is that the linear relationship always results in a perfect fit (*R*^2^ = 1.0). In addition, no parameters for the goodness of fit (i.e., SEE) can be derived. Therefore, it is recommended to use at least three prediction trials to ensure a low standard error for CP (2–5%) and *W*′ (< 10%) (Black et al. 2016; Dekerle et al. 2015). Performing three prediction trials and using a two-parameter CP model to fit the data results in one degree of freedom. For instance, a standard error of 5 W for a cyclist with a CP of 385 W would then need to be multiplied by 12.7 to calculate the 95% confidence limits (± 64 W) in both directions. Adding a fourth prediction trial would reduce the CP standard error to 3 W and the 95% confidence limits (± 38 W) thus improving the CP predictive ability.

The 3-min all out test has also been proposed as a more time efficient way to derive CP and *W*′ (Vanhatalo et al. 2007, 2008). The principal assumption in this test is that *W*′ or more accurately WEP (work above end test power) as it is known in this test, is fully depleted within the first 150 s and therefore during the last 30 s only CP (end test power) can be sustained. Despite showing good reliability and validity compared with traditional CP testing in some circumstances (Wright et al. 2017), other research in elite cyclists shows significantly higher CP estimates are derived from the 3-min all out test than traditional protocols (McClave et al. 2011) which can lead to overestimation of performance capacity in the severe exercise intensity domain (Nicolò et al. 2017). This finding brings into question whether the 3-min all out test can be used in the field of power profiling.

In some power-duration models (see Table 1) *P*_max_ is an additional input parameter when modelling the power-duration relationship. Extensive research (Douglas et al. 2021; Driss and Vandewalle 2013; McCartney et al. 1983, 1985) was conducted on the assessment and mechanisms of *P*_max_ in cycling (Sargeant et al. 1981). Assessing *P*_max_ in the laboratory or field settings requires a thoughtful reflection on testing protocols. Recent research used the highest 1 s power output within 4 s, 10 s and 15 s sprints to derive *P*_max_ (Driss and Vandewalle 2013; Ferguson et al. 2021; Gardner et al. 2007; Sanders and Heijboer 2019b). If efforts longer than 10 s are used *P*_max_ could be negatively influenced as the cyclist may apply a pacing strategy (Driss and Vandewalle 2013; Gardner et al. 2007). Practitioners should also be aware of a “learning effect” during all-out sprint efforts, and it is therefore recommended that adequate familiarization is undertaken prior to formal testing of *P*_max_. Additional important factors to consider when testing *P*_max_ in a laboratory setting are; the torque factor setting (Forbes et al. 2014) and whether the expected *P*_max_ is within the range of validity of the power measuring device. For example, a commercially available smart trainer is only valid up to 700 W, which is much lower than the expected *P*_max_ for some populations (Zadow et al. 2016).

### Ecological validity

Cadence, body position as well as topography, i.e. level ground or uphill conditions, have also been shown to influence model parameter estimates due to different biomechanical recruitment patterns (Bertucci et al. 2005; Kordi et al. 2019; Nimmerichter et al. 2012). Therefore, rider specialization (for example climber vs. time trial specialist) and race demands (uphill vs. flat, on-road vs. off-road, etc.) need to be considered in the selection of testing environments (Nimmerichter et al. 2012). The testing conditions should mirror the conditions in which athletes are expected to perform. For example, it is recommended that time trial specialists perform prediction trials on a time trial bike on level ground, while climbing specialists conduct testing in uphill conditions on a road bike.

Previous research has also investigated whether time trials or TTF trials should be favoured as prediction trials (Coakley and Passfield 2018; Karsten et al. 2018). Traditionally, TTF trials have been based on a fixed percentage (i.e. 80–105%) of the power output in a GXT. The main limitation with this approach being that inter-individual differences could influence the trial duration (Jamnick et al. 2020). In contrast, maximum effort time trialling requires a high level of pacing ability and may therefore only be suitable for use with experienced cyclists (Karsten et al. 2018). However, time trials are inherently easier to perform in field settings, as Simpson and Kordi (2017) have shown a particularly time-effective protocol using time trials in elite athletes can produce valid CP and W´ estimates. However, in less trained participants higher power output values have been reported in TTF trials resulting in higher CP and W´ estimations (Coakley and Passfield 2018).

As mentioned above, environmental factors should be considered whenever performing any formal testing. Testing conditions during formal testing should therefore aim to mirror as closely as possible the competition settings to ensure environmental validity. To illustrate this point, CP has been shown to decline significantly as altitude increases, while *W*′ only decreased above 4.000 m of altitude (Townsend et al. 2017); heat and humidity have been shown to influence power outputs in formal testing (Racinais et al. 2015).

Previous research also investigated the influence of cadence on time trial performance and power-duration parameter estimates. While CP estimates were higher at cadences 60 vs. 100 revolutions per minute in recreationally trained individuals (Barker et al. 2006; Carnevale and Gaesser 1991), no statistically significant differences in physiological determinants (gross efficiency, energy turnover) were reported at cadences between 80 vs. 100 revolutions per minute in elite cyclists during cycling time trials (Foss and Hallén 2005). Although higher power outputs can be achieved at lower cadences, elite cyclists tend to prefer higher cadences around ~ 90 revolutions per minute despite reductions in cycling efficiency.

## Agreement between modelled power-duration relationship and MMP values

Good agreement between CP estimates derived from formal testing and MMP values has been reported (Leo et al. 2020, 2021a; b; Nimmerichter et al. 2020; Quod et al. 2010). While a good agreement between CP derived from formal testing and racing has been shown, the same cannot be confirmed for *W*′. Both Leo et al. (2020) and Karsten et al. (2015) reported low agreement between W´ derived from formal testing and MMP data. This low agreement may be due to cyclists not performing maximal efforts in race situations apart from very specific circumstances (i.e., during time trials or at the finish of races). If cyclists were to fully deplete W´ in any other circumstance (i.e. uphill mountain finish, lead out or tine trial), there is a chance that they may subsequently not be able to match the power requirement to follow the peloton. These scenarios have direct implications on the recorded MMP values thereafter, as they are not as high as the MMP values recorded earlier in the race (Leo et al. 2021b). Thus, these efforts are not being captured via basic MMP analysis per se.

Good agreement has been reported between power outputs predicted by the APR model and race-derived MMP data for short duration power outputs (< 2 min) in professional male cyclists (Sanders et al. 2017; Sanders and Heijboer 2019b). However, only limited research exists to verify if this approach could also be applied to other populations.

## Future directions

Although many approaches concerning power profiling have been developed in the literature, it remains unclear which approach provides the greatest insight. Arguably, the most convenient way for practitioners to create a power profile would be to retrospectively use field derived MMP data from training and racing over pre-defined durations (Ebert et al. 2005; Menaspà et al. 2017; Sanders and van Erp 2021; Vogt et al. 2007b). Although this kind of data may provide valuable insights into racing demands in highly trained cyclists, little information can be retrieved in terms of the power-duration relationship due to the arbitrary selection of MMP values.

Deriving a comparative measure allows longitudinal analysis: for example, if a rider records a MMP value in racing which exceeds the prediction from formal testing, practitioners can use that information to monitor changes in the power profile. However, deriving W´ from racing or field testing has shown poor predictive ability (Karsten et al. 2015; Leo et al. 2021a) questioning the practical utility of *W*′ for power profiling purposes. When creating a theoretical power-duration curve from formal testing, care should be taken that the appropriate models are used. For example, application of the CP concept outside the severe exercise intensity domain involves an overestimation in short MMP (< 2 min) ability and long duration MMP (> 40 min) sustainability. For this reason, the APR model provides a useful concept to predict the power-duration relationship in the extreme exercise intensity domain.

While the power-duration relationship in the severe exercise intensity domain has been well investigated based on the CP concept (Jones et al. 2010; Poole et al. 2016), limited research exists on deriving the power-duration relationship in the moderate and heavy exercise intensity domains (Black et al. 2017). Hence Puchowicz et al. (2020) and Peronnet and Thibault (1989) proposed mathematical models with an aerobic decay term, but limited research exists to assess if these concepts have a high predictive ability for the power-duration relationship in the moderate and heavy exercise intensity domains in relation to the muscle bioenergetic system (Korzeniewski 2019; Korzeniewski and Rossiter 2020, 2021; Vanhatalo et al. 2016).

Recent work (van Erp et al. 2021b; Leo et al. 2021b) has shown a reduction in MMP values as prior work increases. However, future research is needed to better understand the mechanisms which lead to alterations in the power-duration due to fatigue, especially the influence of the exercise intensity and if work in different exercise intensity domains induce the same degree of downward shift in the power-duration curve. This is important as improved performance capacity, i.e. smaller alterations in the power-duration relationship, has been positively related to race success (van Erp et al. 2021b; Leo et al. 2021b).

In the era of big data science a novel approach introduced by Puchowicz (2018) on the Golden Cheetah open data project (Liversedge 2020) could provide novel insights into power profiling. Functional principal component analysis (FPCA) enables an in-depth view of the components of variability in MMP data between cyclists via eigenfunctions which classify riders on their phenotype (sprinter vs. climber) and performance level. Currently, however, the use of FPCA for the purposes of power profiling still requires adequate scientific validation before any potential findings can be applied by coaches and practitioners.

## Practical recommendations in applied settings

Based on the current literature and the authors’ experience conducting power profiling in applied settings, the following recommendations can be made as a starting point for coaches and practitioners: to derive the parameters to model a power-duration curve a formal test protocol should include one sprint effort (i.e. ~ 10–15 s) and at least three maximum efforts between 2 and 15 min (Karsten et al. 2015; Leo et al. 2021a; Muniz-Pumares et al. 2019; Sanders and Heijboer 2019b). These efforts can be completed in a single testing session, though it is recommended to divide field testing into two sessions over two consecutive days. The order of efforts should preferably be randomized for scientific research or follow the cyclist’s or coach’s individual preference in applied settings. Inter-trial recovery between efforts should be set to a minimum of 30 min of active recovery (< 2 rating of perceived exertion) (Karsten et al. 2017). CP and *W*′ should be derived by the non-linear two-parameter CP model (Muniz-Pumares et al. 2019), while *P*_max_ should be referred to the 1 s peak power during the ~ 10–15 s sprint effort (Sanders and Heijboer 2019b). This protocol will allow coaches and practitioners to derive valid *P*_max_, CP and *W*′ estimates. Coaches can then choose the best modelling approach based on the exercise intensity domain(s) that are important for race analysis and training prescription in a given discipline.

Power meters should be verified for accurate and reliable measurement and a zero-offset or re-calibration according to the manufacturer’s recommendations is recommended.

The authors do not recommend using single effort field tests (i.e. 8 min or 20 min TT) to derive the FTP estimate because it lacks physiological background and only represents a single point on the power-duration curve. Nor do they recommend the use of the 3- min all-out test as this may lead to an overestimation of the power-duration relationship in the severe exercise intensity domain.

To increase the ecological validity of power profiling we recommend a careful selection of the power-duration modelling approach, based on biomechanical and physiological principles. Standardized laboratory and field testing should be conducted in line with performance analysis from training and racing to increase the practical utility of performance prediction and training related consequences.

In addition, any formal testing should consider the environmental and topographical conditions in which the power profile information is to be applied in. Therefore, the duration of the effort, gradient, inter-trial recovery, rider type specialization (climbers vs. flat specialist) and race demands (climb vs. time trial) should be replicated as best possible.

Collectively, power profiling provides an advanced opportunity for performance modelling based on power output data from training and racing in combination with traditional laboratory and field-testing methods to maximize cycling performance.

## Data Availability

Not applicable.

## Abbreviations

%$\dot{V}$O_2max_: Fractional utilization of the maximum oxygen uptake
2-P CP: Two-parameter critical power model
3-P CP: Three-parameter critical power model
APR: Anaerobic power reserve
ATP: Adenosine tri phosphate
BMX: Bicycle motocross
CT: Critical torque
CP: Critical power
CP_TTF_: Time to task failure at critical power
*e*: Basis of the natural logarithm (*e* = 2.178)
EVA: Exposure variation analysis
FPCA: Functional principal component analysis
FTP: Functional threshold power
GET: Gas exchange threshold
GXT: Laboratory incremental graded exercise test
*k*: The rate of the exponential decline in power output
LT: Lactate threshold
MAP: Maximum aerobic power
MLSS: Maximum lactate steady state
MMP: Maximal mean power output
OmPD: Omni power duration model
P&T: Peronnet and Thibault model
*P*(*t*): Power output
*P*_max_: Peak power over 1 s
SEE: Standard error of the estimate
TT: Cycling time trial
TTF: Time to task failure
$$\dot{V}$$O_2_: Oxygen uptake
$\dot{V}$O_2max_: Maximum oxygen uptake
*W*′: Work capacity above critical power
WEP: Work above end test power
